# Treatment of Aneurism by Injecting into the Sac a Concentrated Solution of the Perchloride of Iron

**Published:** 1853-11

**Authors:** 


*Treatment of Aneurism by injecting into the sac a concentrated solution of the Perchloride of Iron.*—At a meeting of the London Medical Society, in the early part of April, Mr. Gay read the account of a case (of which the following is an abstract) lately communicated to the Societé de Chirurgie, by M. Larrey, and forwarded to this country by Dr. Costello. In the month of January, Dr. Pravaz of Lyons, made known to the Academie des Sciences a new method of effecting the coagulation of blood, by mixing with it a concentrated solution of the per-chloride of iron; and, at the same time, suggested the employment of this agent for the cure of aneurism. M. Delongchamps, an army surgeon in the same town, followed up the suggestion, and with the happiest results, as the following narrative will show :—

A blacksmith of Le Maus, æt. 26, met with a blow over the left eyebrow. This was followed by an aneurism of the supra-orbital branch of the ophthalmic artery, which gradually increased in size, until it had attained that of a pigeon's egg. Continuous pressure was made over the tumor, by means of a pad and spring, for twenty-five days and nights, but without any benefit; and as the patient was unwilling to submit to the process of electro-puncture, and other remedies did not appear to be suitable to the case, M. Delongchamps decided on trying the proposal of Dr. Pravaz.

On the 4th February, an oblique puncture was accordingly made through the walls of the sac, and whilst compression was made on the artery above and below, a few drops of the solution were injected into it, by means of a small glass syringe with a fine point. A few drops of florid blood followed the withdrawal of the syringe, but without jet; a few more drops of the solution were then injected, and it became at once evident that these had produced the desired effect; for the blood in that portion of the sac into which the nozzle of the syringe had been introduced became rapidly firm, and pulsation ceased. In the remaining portion of the sac, however, no such change took place, which arose from the fact, that a firm plug of coagulum had formed in the orifice of the syringe during the operation, which no force on the piston could dislodge, and prevented the escape of a quantity of the solution sufficient to coagulate the whole of the blood within the sac. The patient suffered no pain, nor did he desist from his ordinary work. On the 6th of February, as the success of former operations had only been partial, ten or twelve drops more were injected, the nozzel of the syringe having been previously well greased, in order to prevent the formation of a plug, as on the former occasion. A sharp burning pain followed, and the contents of the sac could distinctly be felt to be undergoing a process of solidification. Not a drop of blood escaped this time on the withdrawal of the syringe, and pulsation ceased entirely. On the 7th, the patient suffered some throbbing pain and restlessness, and the tumor became swollen and hot. These symptoms, however, subsided quickly under the use of evaporating lotions, etc.; so that, on the 8th, the patient could resume his work. A slight oozing of pus through the last puncture took place a few days afterwards, and with this a subsidence of the tumor. By the 15th of March, the cure was so far advanced, that its situation could scarcely be recognized.

Mr. Gay expressed his opinion, that the treatment of aneurism by the method referred to was deserving of every attention; but that at present its employment should be confined to smaller aneurisms. The affinity of the perchloride of iron for fibrin is so strong, that its coagulation commences as soon as they come into mutual contact; and so firm is the clot that ensues, that no fear need be entertained of any shreds being washed away by the circulating current. As a styptic, this and other experiments that have been made, prove that it is invaluable.—*Edinburg Monthly Journal.*

